# The Potential of AI in Nursing Care: Multicenter Evaluation in Fall Risk Assessment

**DOI:** 10.2196/71034

**Published:** 2025-10-08

**Authors:** Ivana Nanevski, Sebastian Jäger, Matthias Schulte-Althoff, Eva-Maria Behnke, Daniel Fürstenau, Felix Biessmann

**Affiliations:** 1 Berliner Hochschule für Technik Berlin Germany; 2 Department of Information Systems School of Business & Economics Freie Universität Berlin Berlin Germany; 3 Institute of Medical Informatics Charité - Universitätsmedizin Berlin, Corporate member of Freie Universität Berlin and Humboldt Universität zu Berlin Berlin Germany; 4 Medizinische Hochschule Brandenburg Theodor Fontane Neuruppin Germany; 5 Einstein Center Digital Future Berlin Germany

**Keywords:** fall risk prediction, machine learning, artificial intelligence, federated learning, clinical decision support, nursing care, fairness

## Abstract

**Background:**

With 28%-35% of individuals aged 65 years and older experiencing incidents of falling, falls are the second leading cause of unintentional injury–related deaths globally. Limited availability of clinical staff often impedes the timely detection and prevention of potential falls. Advances in artificial intelligence (AI) could complement existing fall risk assessment and help better allocate nursing care resources. Yet, many studies are based on small datasets from a single institution, which can restrict the generalizability of the model, and do not investigate important aspects in AI model development, such as fairness across demographic groups.

**Objective:**

This study aimed to provide a comprehensive empirical evaluation of the potential of AI in nursing care, focusing on the case of fall risk prediction. To account for demographic and contextual differences in fall incidences, we analyze data from a university and a geriatric hospital in Germany. To the best of our knowledge, these are the largest fall risk prediction datasets to date with heterogeneous data distributions. We focus on 3 key objectives. First, does AI help in improving fall risk prediction? Second, how can AI models be trained safely across different hospitals? Finally, are these models fair?

**Methods:**

This study used 2 datasets for fall risk prediction: one from a university hospital with 931,726 participants, 10,442 of whom experienced falls, and another from a geriatric hospital with 12,773 participants, 1728 of whom have fallen. State-of-the-art AI models were trained with 3 approaches, including 2 decentralized learning paradigms. First, separate models were trained on data from each hospital; second, models were retrained on the respective other dataset; and federated learning (FL) was applied to both datasets. The performance of these models was compared with the rule-based systems as implemented in clinical practice for fall risk prediction. Additional analyses were conducted to test for model fairness.

**Results:**

Our findings demonstrate that AI models consistently outperform rule-based systems across all experimental setups, with the area under the receiver operating characteristic curve of 0.735 (90% CI 0.727-0.744) for the geriatric hospital, and 0.926 (90% CI 0.924-0.928) for the university hospital. FL did not improve the fall risk prediction in this setting. Our fairness analysis ruled out disparities in model performance between different sex groups, but we found fairness infringements across age groups.

**Conclusions:**

This study demonstrates that AI models consistently outperform traditional rule-based systems across heterogeneous datasets in predicting fall risk. However, it also reveals the challenges related to demographic shifts and label distribution imbalances, which limited the FL models’ ability to generalize. While the fairness analysis indicated fair results across sex subgroups, age-related disparities emerged. Addressing data imbalances and ensuring broader representation across demographic groups will be crucial for developing more fair and generalizable models.

## Introduction

### Background

Falls are the second most common cause of unintended injury-related deaths worldwide, mostly affecting older adults. Globally, falls are responsible for approximately 680,000 deaths annually [1,2]. Furthermore, falls have been identified as one of the most prevalent risk factors impacting older adults, especially inpatients [3,4]. In the United States, the fall rate is 3.56 per 1000 patient-days [5]. In Germany, an analysis of data from 55 institutions in 2004 reported that the fall rate in hospitals was 4.2 per 1000 patient-days, while in nursing homes, it was 5.1 per 1000 patient-days [6]. According to Rubenstein [7], the average incidence of falls in nursing homes is 1.5 falls per bed per year, with a range from 0.2 to 3.6 falls per bed annually.

The incidence of falls among the older adult population not only causes significant physical health risks, including fractures and head injuries, but also results in psychological and social consequences, such as fear of falling, which can impair quality of life [1,4]. Furthermore, this also leads to increased health care costs and longer hospital stays [5]. The limited availability of professional resources such as physical therapists, nurses, and doctors hinders timely detection and prevention of potential falls [8]. In Germany, nursing care wards are understaffed with an estimated shortage of 100,000 to up to 520,000 nurses by 2030 [9]. According to recent reports, this will continue to increase until 2049 with 280,000-690,000 missing nurses [10]. At the same time, according to World Health Organization global report [2], 28% (782/2793) [11] to 35% (356/1042) [12] of individuals aged 65 years and older experience at least 1 fall, a rate that increases to 50% for those aged 80 years and older. In addition, it is estimated that by 2030, 1 in 6 individuals will be aged 60 years or older [4]. Therefore, it is crucial to develop highly effective assessment tools for predicting fall risk in patients to reduce the number of people who experience falls and to enable nurses to make more informed decisions regarding fall risk management [13,14].

Existing rule-based fall risk assessment tools, such as The Expert Standard for Fall Prophylaxis (ESFP) [15] and the World Guidelines for Falls Prevention (WGFP) [16], are built on existing literature and focus on a broad range of indicators. ESFP focuses on demographic parameters, risk indicators such as fear, fractures, lack of mobility and balance, contracture, diabetes, calcium deficiency, overweight, depression, mobility aid (such as walking stick, wheelchair, or knee tutor), dementia or cognitive impairment, and the Timed “Up and Go” (TUG) test [17]. WGFP is based on a set of rules that cover (1) mobility-related risk factors such as the fear of falling; (2) mobility aid such as walking stick, wheelchair, or knee tutor; (3) the TUG test; (4) sensory functioning indicators such as dizziness, glasses, acuity, contract perception, hearing aid; (5) the Barthel index [18]—a measurement for activities of daily life; (6) the cognitive function rules, such as the Mini-Mental State Examination (MMSE) [19], (7) presence of delirium; (8) behavioral patterns such as excitement, focus, apathy, tendency to stray; (9) autonomous functions such as orthostatic hypotension, nocturia or incontinence; (10) medical history such as signs of Parkinson disease or depression; and (11) nutrition history and vitamin D deficiency.

In contrast to these guidelines using a broad range of indicators, others often focus on a smaller set of indicators, for example, the Morse Fall Scale [20], the Hendrich II Fall Risk Model [21], and the St Thomas Risk Assessment Tool [22]. Often, those who use specialized tests, such as TUG or Tinetti [23], evaluate whether patients have balance or walk impairments. In addition to traditional risk assessment tools, there have been attempts to develop machine learning (ML) and artificial intelligence (AI; ML and AI have been used interchangeably) models for better fall risk assessment [3-5,8,14,24-32]. Multiple studies leverage data from wearable devices [8,24,25] and camera motion tracking data [29,31] for fall prediction. For example, Shu and Shu [31] use a camcorder to gather data, and they base their analysis on different types of falls. Other studies focus on tabular data [3,4,14,28,30,32,33] such as demographic information, initial diagnosis, risk factors (such as history of falls), activities of daily living, medication, or cognitive impairments. The most commonly used models for tabular data in these studies were support vector machines, logistic regression, decision trees, random forest, and gradient boosting trees. Of these, random forests and gradient boosting trees have been shown to perform better on tabular data [34].

However, these studies rely on data from a single hospital, which could restrict the models’ ability to generalize the datasets from other hospitals. These generalization issues stem from fundamental demographic differences between datasets and emphasize the importance of inclusive data collection, as different distributions often carry distinct risk factors [27,35]. Achieving broader population representation for training AI models can be challenging, particularly in hospital settings, where data privacy is important. However, there are potential solutions, such as retraining of models on local data [36], federated learning (FL) [37], and variations thereof [38]. In such decentralized learning schemes, multiple participants or institutions simultaneously train a common AI model. FL often shows advantages in the medical domain, addressing key challenges such as data privacy, data scarcity, and the need for collaborative research. Numerous studies [39-42] have shown that FL can help in developing joint models by leveraging data from multiple centers.

### Objective

In this study, we use 2 large-scale tabular datasets, coming from one of the largest hospitals of Germany and from a smaller geriatric hospital. We explore the potential of AI in addressing one of the key challenges in modern health care systems: developing data-driven AI models which can handle heterogeneous data distributions, such as those arising from age variations. Our study focuses on 3 primary research objectives. First, we examine whether AI models can provide improvements over traditional rule-based fall risk assessment tools used in nursing care. Second, we explore approaches for training AI models to collaborate on 2 large-scale datasets. Finally, we assess if the models are fair across diverse demographic groups. To the best of our knowledge, this study represents the most comprehensive evaluation on the potential of ML for fall risk assessment in terms of data size, and no existing related studies have specifically focused on investigating the fairness aspect of a fall prediction model on such a large-scale dataset in Germany.

## Methods

### Datasets

#### General Data Overview

We used 2 anonymized datasets extracted from a large university hospital and a geriatric hospital in Germany. Both datasets contain central demographic information (age and sex), diagnoses, procedures, and fall risk assessment scores obtained from tools like the TUG test or the Jones Index [43]. The procedures, which are interventions performed by a health care professional to diagnose, treat, monitor, or prevent a health condition, follow the standardized “German Operationen- und Prozedurenschlüssel (OPS)” [44]*.* The diagnoses follow the *ICD-10-GM* (*International Statistical Classification of Diseases, Tenth Revision, German Modification*) [45], representing the patients’ most common diagnosis in their electronic health records (EHRs).

#### ICD and OPS

ICD (International Classification of Diseases) and OPS are hierarchical classification systems. For example, ICD I21.4 encodes “acute subendocardial myocardial infarction,” which belongs to “acute myocardial infarction” (I24). This broader category is itself part of “ischemic heart diseases” (I20 to I25) that fall under the umbrella of “diseases of the circulatory system” (I00 to I99). Preliminary experiments with different levels showed best results when using the fourth level (ie, I21.4) if available, otherwise level 3 (Z11). Similarly, for OPS, we use the third level. For example, “diagnostic catheter examination of the heart and circulation” (1-27) belongs to “examination of individual body systems” (1-20 to 1-33), which is part of “Diagnostic Measures” (1-10 to 1-99).

#### Fall Risk Assessments

Fall risk assessments are conducted manually by nursing staff during the admission process after hospitalization and updated regularly (at least every 5 days). The goal is to gather information to determine whether patients have an increased risk of falling to apply preventive measures. Both hospitals’ assessments are motivated by ESFP and WGFP described in previous sections. Therefore, they similarly ask for past fall incidences, mobility impairments, cognitive impairments, excretions, and certain medications, such as psychotropic drugs or sedatives. However, they use different measurements to determine patients’ impairments.

The university hospital adheres to the patient classification system introduced by Jones [43], which assesses the patients’ independence in different dimensions of daily life, such as feeding, personal toilet, and walking. On the other hand, the geriatric hospital employs a suite of measurements specialized for specific aspects. The TUG test measures the time (in seconds) required for a patient to stand up from a chair, walk 3 meters, turn, walk back, and sit down again. A TUG score of ≥20 seconds is indicative of a mobility impairment. The MMSE [19] test is a series of questions and verbal and written commands to test for cognitive impairments. Patients can achieve up to 30 points (more is better), where MMSE ≤23 is considered a cognitive impairment. Finally, the Tinetti test assesses patients’ balance (15 points) and gait (13 points) to determine their risk of falling. A score of ≤20 (out of 28) points is indicative of an increased fall risk.

#### University Hospital Data

The university hospital dataset comprises 931,726 EHRs ranging between 2016 and 2022. The patients range in age from 19 to 114 (mean 58, SD 19.99) years and a median value of 59 (IQR 39-74) years. About (496,560/931,726, 53.3%) of the patients are female. A mere (10,442/931,726, 1.12%) of patients have experienced at least 1 fall incident, which occurs more often for older patients (Table 1). In this dataset, there is only 1 entry per patient.

**Table 1:** Patient distribution and fall prevalence (overall, by sex and by age). Counts and percentages are shown for all patients, fallers, and nonfallers.

Dataset and characteristics				All patients, n (%)		Fallers, n (%)		Nonfallers, n (%)
University								
	Patients			931,726 (100)		10,442 (1.12)		921,284 (98.88)
	Sex							
		Female	496,560 (100)		4696 (0.95)		491,864 (99.05)	
		Male	435,166 (100)		5746 (1.32)		429,420 (98.68)	
	Age range							
		10-20	3349 (100)		3 (0.09)		3346 (99.91)	
		20-30	83,926 (100)		144 (0.17)		83,782 (99.83)	
		30-40	155,611 (100)		316 (0.2)		155,295 (99.8)	
		40-50	142,956 (100)		427 (0.3)		142,529 (99.7)	
		50-60	144,232 (100)		846 (0.59)		143,386 (99.41)	
		60-70	135,627 (100)		1577 (1.16)		134,050 (98.84)	
		70-80	118,549 (100)		2366 (2)		116,183 (98)	
		80-90	100,742 (100)		3061 (3.04)		97,681 (96.96)	
		90-100	40,067 (100)		1449 (3.62)		38,618 (96.38)	
		100-110	6445 (100)		249 (3.86)		6196 (96.14)	
		110-120	222 (100)		4 (1.8)		218 (98.2)	
Geriatric								
	Patients			12,773 (100)		1728 (13.53)		11,045 (86.47)
	Sex							
		Female	7304 (100)		866 (11.86)		6438 (88.14)	
		Male	5469 (100)		862 (15.76)		4607 (84.24)	
	Age range							
		60-70	1281 (100)		145 (11.32)		1136 (88.68)	
		70-80	4560 (100)		551 (12.08)		4009 (87.92)	
		80-90	5851 (100)		852 (14.56)		4999 (85.44)	
		90-100	1069 (100)		177 (16.56)		892 (83.44)	
		100-110	12 (100)		3 (25)		9 (75)	

#### Geriatric Hospital Data

This dataset is considerably smaller, with only 12,773 patients between 2019 and 2022. Furthermore, the prevalence of fall incidence is higher (1728/12,773, 13.53%; Table 1), and the patients are older, ranging between 62 and 102 (mean 79, SD 7.23) years, and a median value of 80 (IQR 75-85) years. About (7304/12,773, 57.2%) of the patients are female. In addition, this dataset includes a measure of the patients’ independence, the Barthel index [18]. The index considers various daily activities, such as feeding, personal toilet, walking, and so on, and computes a score between 0 and 100, where 100 indicates full independence and 0 indicates full dependence on help. In this dataset there is only 1 entry per patient.

#### Common Data Schema

In this study, we encountered the challenge of integrating data from 2 different sources, the geriatric hospital and university hospital, which initially had distinct data schemas. To develop a comprehensive and effective prediction model, it was crucial to harmonize these datasets into a unified format. This process involved identifying common features between the 2 hospitals’ data schemas and aligning them accordingly. To achieve a unified dataset schema, we mapped the common features across both hospitals, ensuring that each corresponding attribute holds the same type of information and adheres to the same format. For example, the indicator if a person is wearing an orthosis in one dataset is listed under “medical items,” and in the other under “mobility,” both defined as text fields. We mapped both columns to a common binary feature. For the features unique to either geriatric or university hospitals, we incorporated them into the combined schema by assigning null values where the data are not available. This approach ensures that no information is lost while preserving the dataset’s integrity. The common data schema consisted of 124 columns (Table S1 in Multimedia Appendix 1). By carefully managing the disparities in data schemas, we ensured that our fall prediction model could perform well across both hospital environments.

#### Ethical Considerations

The ethics committee of the Charité – Universitätsmedizin Berlin, Germany, approved this data analysis (EA2/184/21). Due to the retrospective design of the study using data from standard care, the need for informed consent was waived. The data protection officers of the Charité and the Evangelische Geriatriezentrum Berlin advised on data protection rules to ensure compliance with those rules. Data from the Charité were anonymized using standard procedures involving their Health Data Platform, while the Evangelische Geriatriezentrum Berlin anonymized their data according to internal procedures before analysis. We consulted the guidelines for developing and reporting ML predictive models in biomedical research [46].

### Experiments

#### Baselines

**ESFP**

The ESFP is curated by the German Network for Quality in Nursing [15] and the German Board of Trustees for the Elderly [47]. The aim of the ESFP is to determine the risk of falls for people in need based on a selection of risk factors. These factors include the history of falls and fractures, fear of falling, mobility impairment (strength, balance, endurance, and flexibility), and cognitive impairments. They recognize three additional types of risk factors: (1) personal fall risk factors, such as vision impairment or cognitive impairment; (2) medication-related fall risk factors, such as psychotropic drugs that affect mental processes; and (3) environmental fall risk factors, for example, obstacles on the floor. This guideline is used by nurses who should assess patients for all potential risk factors and note their severity. Patients who are at high risk of falling should receive help that prevents falls.

**WGFP**

The WGFP [16] is the product of a worldwide collaboration of 96 experts. While developing the guidelines, they incorporated input from older adults, and final decisions were reached through a minimal voter consensus. Initially, the guidelines have 2 ways of assessing the fall risk. First, there is the opportunistic case finding which includes people who fell in the last 12 months. This approach has low sensitivity as it does not take any risk factors into account but can be used for certain patients, for whom more information is not available. The other, more recommended tool from the guideline, applies to patients who have fallen in the past year, or feel unsteady when standing or walking, or worry about falling. Then, an additional assessment identifies the risk of falling. The overall assessment is rule-based, and the end outcome is the indicator if a patient needs additional help. Its decisions are based on some risk factors, such as injury or loss of consciousness, as well as some directly measured values, like a TUG test resulting in more than 15 seconds.

**Implementation**

We implemented these rule-based systems using the available data (described in Datasets section). If a required parameter for a rule was missing in the datasets, that rule was excluded. To estimate the probability of falling, we calculated the percentage of applicable rules that were fulfilled for each patient. For certain parameters, such as diabetes and depression, we determined presence by checking whether the patient had associated *ICD* codes. An example rule would then be: if a patient has calcium deficiency, then the risk for fall increases. The parameters used for implementing ESFP and WGFP are listed in Tables S2 and S3 in Multimedia Appendix 1, respectively.

#### AI Model and Experimental Frameworks

##### AI Model

Random forests and gradient boosting trees are recognized for their performance on tabular data, as evidenced by recent research [34]. Among these techniques, Extreme Gradient Boosting (XGBoost) has distinguished itself as a particularly effective algorithm for handling tabular datasets, especially in the medical domain [3,28]. In our study, initial experiments indicated that tree-based models outperform other model classes (Table S9 in Multimedia Appendix 1). Therefore, in this study, we chose XGBoost as our AI model and its python implementation [48] accordingly.

##### FL

FL [37] is a collaborative learning approach wherein multiple participants or institutions simultaneously train a ML model. Instead of collecting training data in a central repository, the sensitive data stays within the respective institution and instead of the data, model parameters are shared among institutions, thus maintaining data privacy. This approach is particularly advantageous when individual institutions lack sufficient data to independently train a robust model. Each participant then sends their model updates to a central server to aggregate them and create a unified common model. The unified model is then sent back to all participants for additional training. FL often shows advantages in the medical domain, addressing key challenges such as data privacy, data scarcity, and the need for collaborative research. Numerous studies [39-42] have shown that FL can help in developing joint models by leveraging data from multiple medical institutions. For our FL experiments, we used Flower [49], a community-driven python tool that supports XGBoost. Flower facilitates FL by enabling multiple clients to collaboratively train a model without sharing their raw data. We chose this tool because of community support and the ease of implementing such a complex framework.

### Experimental Setup

#### Baselines

We included the rule-based decision systems WGFP and ESFP as baselines against which we benchmarked our models. As our first key objective included comparison with these rule-based models, we applied them to each dataset to assess whether AI models can provide improvements over the established rule-based fall risk assessment tools for nursing care.

#### Separate Models

In this setting, one model for each dataset was trained exclusively on that data, providing a baseline for hospital-specific performance. The reason behind these sets of experiments was twofold. First, we wanted to compare each hospital individually with the baselines and conclude the assumptions in the first objective. Second, with this experiment, we partially focused on our second objective, that is, evaluating decentralized approaches for training AI models across multiple institutions. These results served as a baseline comparison for the more common centralized training approach for training AI models. It is important to acknowledge that in a setting where hospitals do not collaborate with each other, this is the only viable experiment on a single-institution basis. We train 2 separate models, one for each dataset—geriatric and university. In these experiments, each model is trained using the common data schema. In an early round of our experiments, we verified that the common schema does not affect the models’ outcomes, compared with the initial results where each model was trained using the specific schema of its respective dataset. We will hereinafter refer to these models as “separate.”

#### Retrained Models

Initially trained on data from one hospital, each of the 2 models were subsequently retrained using data from the other hospital, allowing us to assess the benefits of knowledge transfer between hospitals. This approach is also referred to as swarm learning [38] and is particularly useful when notable domain shifts exist between institutions, as it allows models to better represent a broader, real-world population. The idea behind this experiment was to gain intuition on how 2 different large-scale hospital datasets, with considerable demographic shifts within the age distributions, can complement each other’s knowledge. To conduct these experiments, we apply the common data schema to ensure that the models operate consistently across both datasets. For retraining on the second dataset, we used the same model and parameters obtained during the separate training phase on the first dataset. We initialized the model with those parameters and continued training on the other dataset, following the same procedure as in the separate experiments. We performed the retraining twice, once with the separate model trained on the geriatric hospital, and then retraining it on the university hospital data, and the other way around. With this experiment, we partially focused on our second objective and showed an approach for collaboration of 2 large scale datasets: is FL more advantageous than other decentralized approaches? We will refer to these experiments as “retrained.”

#### FL Model

This model was trained simultaneously on data from both hospitals. As detailed in the FL section, FL is a collaborative model training across multiple institutions while preserving privacy, which allows models to benefit from diverse data sources. We performed this experiment to determine whether combining these 2 different large-scale datasets, following the common schema, could contribute to a more effective model by leveraging the collective knowledge from both institutions. We will refer to this model as “federated.”

#### Data and Models Environment

The experiments were conducted using a single machine to simulate a distributed environment. For the retrained experiments, the model was initially trained on 1 dataset and then saved to disk. Subsequently, the model was loaded from disk, along with a second dataset, and further trained. In a practical distributed setting, instead of saving the model to disk, it would be encrypted and digitally transferred to another hospital or accessed via a shared infrastructure. For the FL experiments, a central server managed the secure distribution of models to participating clients. Again, all 3 processes were run on a single machine, with 2 clients simulating hospitals, each accessing a single dataset and communicating solely with the central server. This single-machine simulation is functionally equivalent to a distributed system with machines communicating over the internet but reduced security risks for the institutions participating in this study.

#### Cross-Validation

To determine the optimal set of hyperparameters in each experiment, we conducted a random search [50] for each dataset, selecting certain ranges for each hyperparameter (Table 2). We performed a nested k-fold cross-validation, with k=5, to optimize for the best set of hyperparameters, consisting of: a learning rate (eta) of 0.0089, 97 parallel trees (num_parallel_tree), maximum depth (max_depth) of 5 for each tree, the minimum child weight (min_child_weight) was set to 3, and the 57.8% (0.578) of all features were used by each tree (colsample_bytree). These parameters provided the best results across both institutions. In the outer loop of our k-fold cross-validation (k=5), we evaluated the models’ generalization and robustness by comparing the test set scores over every fold. In contrast to other studies [5,30,51], we trained and tested on stratified splits derived from the original dataset distribution. Using the initially chosen hyperparameters (Table 2), we also performed a k-fold cross-validation (k=5) in the retrained and federated experiments in order to evaluate the models’ generalization by looking at the results of all 5 test folds. We did not remove negative samples from the distribution (undersampling) nor performed oversampling, and worked with the original imbalanced class distribution.

**Table 2:** Artificial intelligence model—range of hyperparameter values.

Parameter	Range
num_parallel_tree	10 to 210
eta	5e-3 to 0.2
max_depth	3 to 11
colsample_bytree	0.5 to 1
min_child_weight	1 to 6

### Evaluation Metrics

In our model evaluation strategy, we aimed at comparability with previous work and evaluated all relevant metrics, but focused primarily on the established metrics. We chose area under the receiver operating characteristic curve (AUROC) for optimization and model evaluation for comparability with previous studies on fall risk assessment [3-5]. As AUROC can be difficult to interpret in the presence of heavily imbalanced class distributions [52], we also provide precision-recall (PR) plots to better assess the classification performance. Given that our models were optimized for the highest AUROC score, we needed a calibrated threshold and a better-suited metric for the binary decisions when directly comparing the models. To perform a calibrated evaluation, we selected a fixed decision threshold based on the *F*_1_-score, since it is a combined metric of both precision and recall, which was further used for fairness analysis.

### Fairness Metrics

#### Equal Opportunity

While there is a sizeable number of fairness metrics available [53], in this study we focused on the most established ones [54]. Equal opportunity specifically focuses on ensuring that the false negative rate (FNR) is equal across the different groups. The FNR is the probability that a participant in the positive class is incorrectly predicted to be in the negative class [54]. Equal opportunity is measured as:



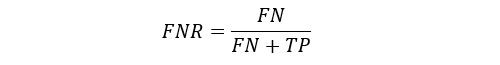



where FN is the number of false negatives and TP is the number of true positives. For equal opportunity to be satisfied, we need:

FNR_Group A_=FNR_Group B_

Mathematically, a classifier with equal FNR will also have equal true positive rate, which directly translates into recall.

To give an example of how equal opportunity is relevant for this study, consider 2 patients in our geriatric ward: one is 70 years old female with a high risk of falling, and the other is 70 years old male with the same risk level. Suppose the predictive model has missed that the female patient is at high risk of falling, and thus this patient does not get timely intervention. Equal opportunity then requires that the model should similarly estimate that the male patient should not be at risk, thus a potential sex inequality would be excluded from the model in this scenario.

#### Predictive Parity

Predictive parity examines whether the probability that a participant with a positive predictive value (PPV) truly belongs to the positive class (or precision) is equal across different subgroups [54]. The precision is measured as:



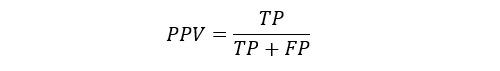



where TP is the number of true positives and FP is the number of false positives. For predictive parity to hold, we need:

PPV_Group A_=PPV_Group B_

This metric is also referred to as precision. Mathematically, a classifier with equal PPV will also have an equal false discovery rate (FDR). To explain how predictive parity would be relevant in this setting, consider 2 patients in our geriatric ward: one is 70 years old with a high risk of falling, and the other is 90 years old with the same risk level. Suppose the predictive model has identified that the 90-year-old patient is at high risk of falling, and thus this patient needs additional preventive measures. Predictive parity then requires that the model should similarly identify the 70-year-old patient as high risk, so this patient would not be excluded from intervention, irrespective of their age.

#### Evaluating Fairness

To directly assess whether a model satisfies predictive parity or equal opportunity, we compare the relevant metrics across demographic groups by computing pairwise ratios of their scores. Consider, for example, 3 age groups: 50-60 years, 60-70 years, and 70-80 years. To evaluate predictive parity, we compute the PPV for each group and derive pairwise ratios such as (PPV_50-60_/PPV_70-80_), (PPV_50-60_/PPV_70-80_), and (PPV_60-70_ /PPV_70-80_). According to the commonly used 80% rule, these ratios should lie within the interval (0.8-1.25) to indicate fairness [55].

However, this ratio-based fairness evaluation becomes unstable when the underlying PPV values are small, which is often the case under severe class imbalance. Since the ratio function is more sensitive near zero, even small absolute differences in the PPV values can lead to large relative disparities, potentially resulting in fairness infringement. To mitigate this instability, we instead focus on the FDR (FDR=1–PPV) for predictive parity and the FNR (FNR=1–TPR), where TPR stands represents true positive rate, for equal opportunity, since these tend to have larger values under class imbalance and thus yield more robust estimates of fairness indicators.

### Feature Importance

To investigate potential causes for the difference in predictive performance between the established rule-based systems and AI models, we used Shapley Additive Explanations (SHAP) to find the most important features for our models. It is a commonly used approach among researchers for interpreting AI models [3,14]. SHAP calculates the average marginal contribution of each feature by considering all possible combinations of features, providing a comprehensive measure of each feature’s influence on the prediction. It is especially important in clinical settings, ensuring that the AI model decisions are explainable. In this study, we used shap [56], a widely used Python implementation for SHAP analysis.

## Results

### AI Models and FL Outperform the Rule-Based Models in Fall Risk Prediction

**Figure 1:**
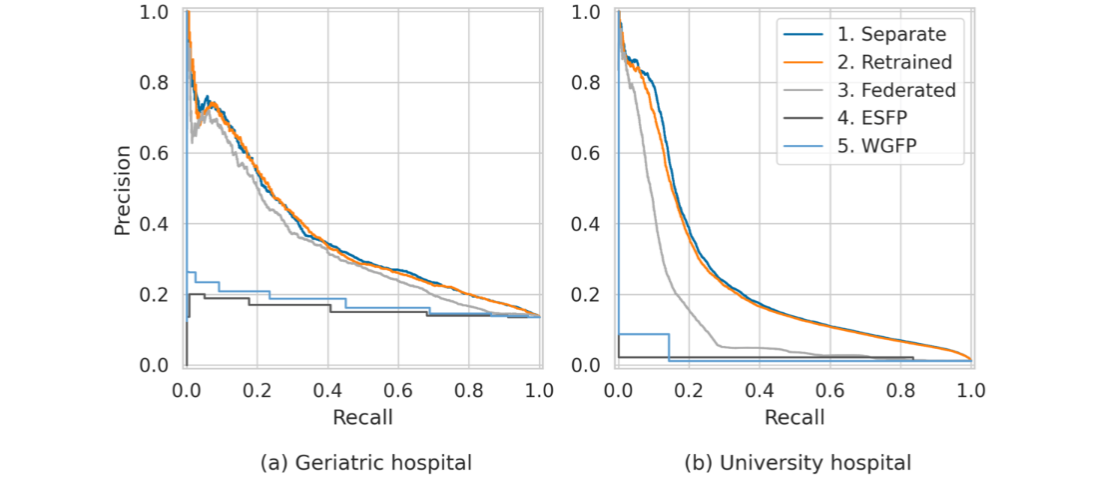
The artificial intelligence–based models (separate, retrained, and federated) substantially outperform the baseline rule-based models (Expert Standard for Fall Prophylaxis [ESFP] and World Guidelines for Falls Prevention [WGFP]). Federated learning (gray curves) struggles to effectively integrate knowledge from the 2 hospitals with considerable demographic shifts, leading to reduced performance for the university hospital.

The PR curves (Figure 1) illustrate the performance of all 3 experiments conducted on both datasets. For both hospitals, the areas under the PR curves indicate that the AI models achieve a substantial improvement over the baselines, and in the case of the university hospital the baseline models do not yield any positive results. For the geriatric hospital (Figure 1A), all 3 learning approaches (separate, retrained, and federated) achieve similar results. In the case of the geriatric hospital, FL does not improve predictive performance. For the university hospital (Figure 1B), there is a notable decline in performance in the decentralized FL setting, suggesting that the federated approach disadvantages this hospital. Similarly, the retrained models also fail to improve performance over the separate models.

To further investigate this, we also compare the models’ AUROC scores (Table 3). Based on the distributions of performance scores across the 5 cross-validation folds, we computed medians and 90% CIs (5th and 95th percentiles) for each of the 3 experiments: separate, retrained, and federated. For the geriatric hospital, we observe no improvement over the separate model from the other models. In contrast, FL results in a decrease in the AUROC score for the university hospital, dropping from 0.926 with the separate model to 0.841. Overall, our findings indicate that retraining and FL can result in models “unlearning” crucial information at the hospital level, thereby failing to outperform the separate models. In this setting, FL does not offer advantages over retraining models either. This indicates that FL is unable to effectively leverage the knowledge from the 2 hospitals, which have considerably different demographic distributions. Consequently, we continue our analysis based on the separate models.

**Table 3:** Median AUROC scores and 90% CI (5th and 95th percentiles): FL fails to enhance performance, with a notable decrease in area under the receiver operating characteristic curve scores at the university hospital from 0̃.926 and 0.924 (Separate and Retrained models) to 0.841, and no improvement over the Separate and Retrained models for the geriatric hospital.

Model	Geriatric hospital	University hospital
ESFP^a^	0.556 (0.533-0.598)	0.703 (0.702-0.707)
WGFP^b^	0.606 (0.572-0.608)	0.565 (0.559-0.565)
Separate	0.735 (0.727-0.744)	0.926 (0.924-0.928)
Retrained	0.735 (0.722-0.741)	0.924 (0.922-0.927)
Federated	0.737 (0.723-0.747)	0.841 (0.820-0.843)

^a^ESFP: Expert Standard for Fall Prophylaxis.

^b^WGFP: World Guidelines for Falls Prevention.

For a calibrated evaluation and for the fairness analysis, we select a fixed decision threshold based on the *F*_1_-score, a combination of precision and recall. Our analysis confirms that the AI models consistently outperform the rule-based models (Table S4 and S5 in Multimedia Appendix 1).

### Fairness Analysis of Fall Prediction

#### Evaluating Fairness

To assess fairness across demographics (sex and age), we examine predictive parity (related to precision) and equal opportunity (related to recall) as illustrated in (Table 4 and 5). For each demographic group within both datasets, we use FDR and FNR to assess predictive parity and equal opportunity, respectively. Fairness is evaluated by calculating the ratio of scores between 2 groups within each demographic. A ratio of 1 denotes equal predictive performance of the AI model between groups. To better interpret the numbers and define a fair outcome, we refer to the 80% rule [55], which considers a ratio within (0.8-1.25) as fair. In the AI fairness literature, there is usually one protected reference group within a given demographic, against which one would compare the scores and discuss fairness [54]. In our experiments, we select the majority group within every demographic of a single hospital to be the reference group (Table 4 and 5). For a more fine-grained analysis, we also computed fairness metrics for all pairs of demographics in each hospital (Figure S1 in Multimedia Appendix 1) and applied a Wilcoxon signed-rank test for both age and sex subgroups (Tables S6, S7, and S8 in Multimedia Appendix 1).

**Table 4:** Geriatric hospital – fairness ratios (false negative rate and false discovery rate) relative to the reference groups, female for sex and 80-90 years for age, are assessed using the 80% rule, where values within (0.8-1.25) are fair. Median false negative rate and false discovery rate ratio scores and 90% CI (5th and 95th percentiles): Sex – the artificial intelligence model is fair – achieves both equal opportunity (false negative rate ratio) and predictive parity (false discovery rate ratio). Age range – in comparison to the reference group (80-90 y), the artificial intelligence model is fair for all patients aged 60-70 y, 70-80 y, and 90-100 y, but not fair for patients aged 100-110 y.

Feature and group		FNR^a^ ratio (90% CI)	FDR^b^ ratio (90% CI)
Sex			
	Male	0.870 (0.687-0.980)	0.912 (0.858-0.981)
	Female^c^	1.000 (1.000-1.000)	1.000 (1.000-1.000)
Age range (y)			
	60-70	1.103 (1.058-1.368)	0.915 (0.688-1.177)
	70-80	1.140 (1.045-1.206)	1.035 (0.979-1.132)
	80-90^c^	1.000 (1.000-1.000)	1.000 (1.000-1.000)
	90-100	1.048 (1.014-1.116)	1.129 (0.966-1.185)
	100-110	1.593 (1.482-1.699)	1.619 (1.547-1.784)

^a^FNR: false negative rate.

^b^FDR: false discovery rate.

^c^Reference group.

**Table 5:** University hospital – fairness ratios (false negative rate and false discovery rate) relative to the reference groups, female for sex and 30-40 years for age, are assessed using the 80% rule, where values within (0.8-1.25) are fair. Median false negative rate and false discovery rate ratio scores and 90% CI (5th and 95th percentiles): Sex – the artificial intelligence model is fair – achieves both equal opportunity (false negative rate ratio) and predictive parity (false discovery rate ratio). Age range - the artificial intelligence model shows fairness infringements for patients aged 40-120 y for predictive parity (false discovery rate ratio) and no fairness infringements for equal opportunity (false negative rate ratio).

Feature and group		FNR^a^ ratio (90% CI)		FDR^b^ ratio (90% CI)
Sex				
	Male	0.957 (0.937-0.983)	1.005 (0.969-1.038)	
	Female^c^	1.000 (1.000-1.000)	1.000 (1.000-1.000)	
Age range (y)				
	10-20	0.849 (0.556-1.093)	0.000 (0.000-0.000)	
	20-30	0.964 (0.846-1.080)	0.857 (0.304-1.396)	
	30-40^c^	1.000 (1.000-1.000)	1.000 (1.000-1.000)	
	40-50	0.946 (0.882-1.031)	1.273 (0.654-1.527)	
	50-60	0.899 (0.890-0.976)	1.421 (0.959-1.544)	
	60-70	0.862 (0.816-0.898)	1.440 (0.961-1.607)	
	70-80	0.843 (0.780-0.873)	1.448 (0.931-1.686)	
	80-90	0.830 (0.771-0.849)	1.454 (0.947-1.630)	
	90-100	0.806 (0.755-0.839)	1.391 (0.957-1.585)	
	100-110	0.821 (0.781-0.900)	1.366 (0.954-1.582)	
	110-120	1.132 (1.126-1.185)	2.000 (1.319-2.280)	

^a^FNR: false negative rate.

^b^FDR: false discovery rate.

^c^Reference Group.

#### General Findings

Our results indicate that AI model predictions are fair with respect to sex across both predictive parity and equal opportunity on both datasets (Table 4 and Table 5). In terms of age, the models are generally fair, although some disparities are observed between the reference and protected groups in both datasets. We note that the interpretation of the results depends to some extent on the fairness definition applied in the analysis. When comparing all pairs of subgroups in a Wilcoxon signed-rank test for both age and sex subgroups (Tables S6, S7, and S8 in Multimedia Appendix 1) there is no statistically significant difference between groups, suggesting that the AI models are fair. However, when applying the 80% rule, our results suggest fairness infringements across age groups in some cases (Table 4 and 5).

#### Fairness Analysis in the Geriatric Hospital

To interpret the fairness results for the geriatric hospital, we use the majority group within each demographic as the reference: 80-90 years for age and female for sex. Fairness scores for the other groups are then computed relative to these reference values (Table 4). Groups which have fair scores in comparison with the reference group have ratio values within the fairness range (0.8-1.25). The model shows fairness across both predictive parity and equal opportunity for sex. In terms of age, the model is generally fair across almost all age groups when compared with the reference group. Disparities are observed for patients aged 100-110 years in both predictive parity (FDR) and equal opportunity (FNR), with this group showing higher values for both metrics. Our results show that in comparison with the reference group, the AI model wrongly assigns high fall risk to many patients aged 100-110 years, while it wrongly assigns low fall risk to other patients aged 100-110 years.

#### Fairness Analysis in the University Hospital

To interpret the fairness results for the university hospital, we use the majority group within each demographic as the reference: 30-40 years for age and female for sex. Fairness scores for the other groups are then computed relative to these reference values (Table 5). Groups which have fair scores in comparison with the reference group have ratio values within the fairness range (0.8-1.25). The results suggest that the model predictions are fair across both predictive parity and equal opportunity for sex. In terms of age, the model is generally fair across most age groups when compared to the reference group. However, patients aged 40-120 years show disparity in predictive parity (FDR), with higher FDR scores in comparison with the reference group, suggesting that the reference group can better compensate for their risk. In contrast, patients aged 10-20 years have a 0 FDR score. However, no fairness infringements were found in the equal opportunity assessment.

### Feature Importance

#### General Findings

**Figure 2:**
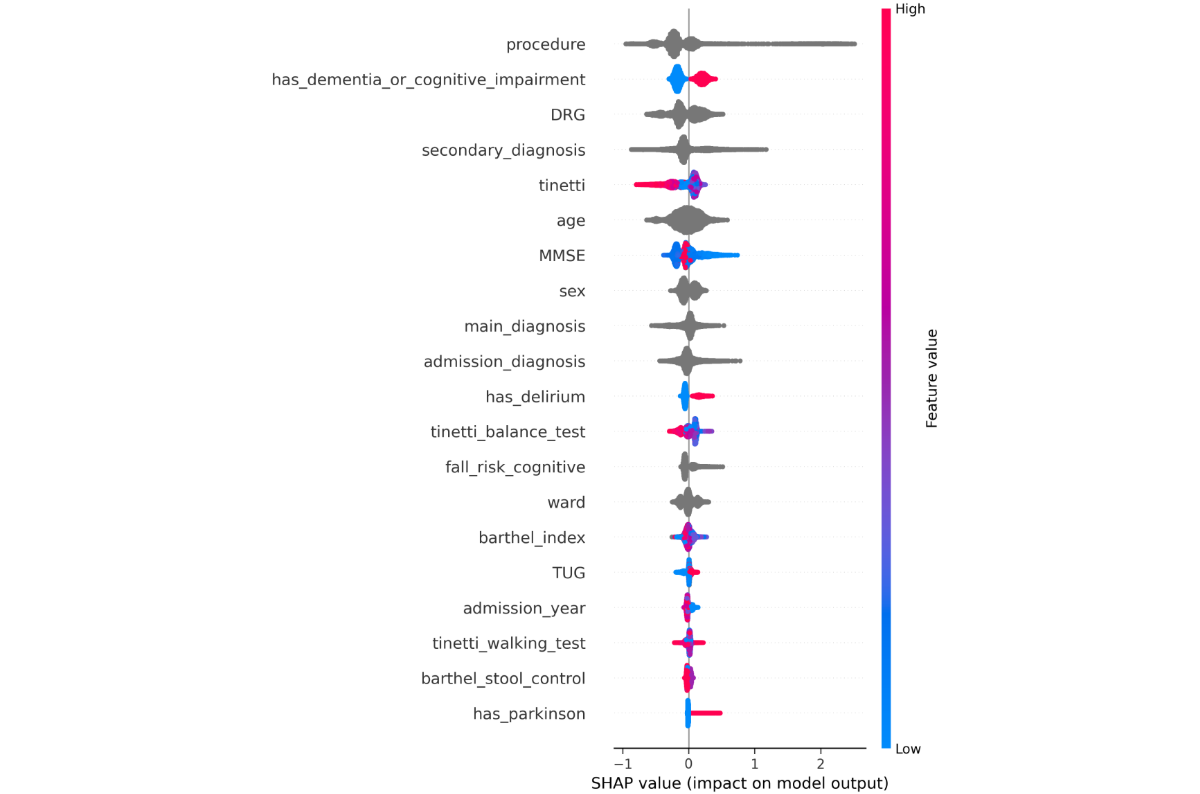
Geriatric hospital—each point along the x-axis represents a single patient and indicates its Shapley Additive Explanations value for that feature. The Shapley Additive Explanations values are used to determine feature importance, with features listed in descending order of their contribution to the model’s predictions. Red color means that the feature has a higher value, and a blue color means that the feature has a lower value. Categorical features are shown as gray points. The most important features include procedure, has_dementia_or_cognitive_impairment, the billable diagnosis code (DRG), secondary_diagnosis, age, the Tinetti score, and Mini-Mental State Examination. Individuals with dementia or cognitive impairment face a higher fall risk (red points along the x-axis). Patients with higher Tinetti scores are mostly at a lower risk of falling.

**Figure 3:**
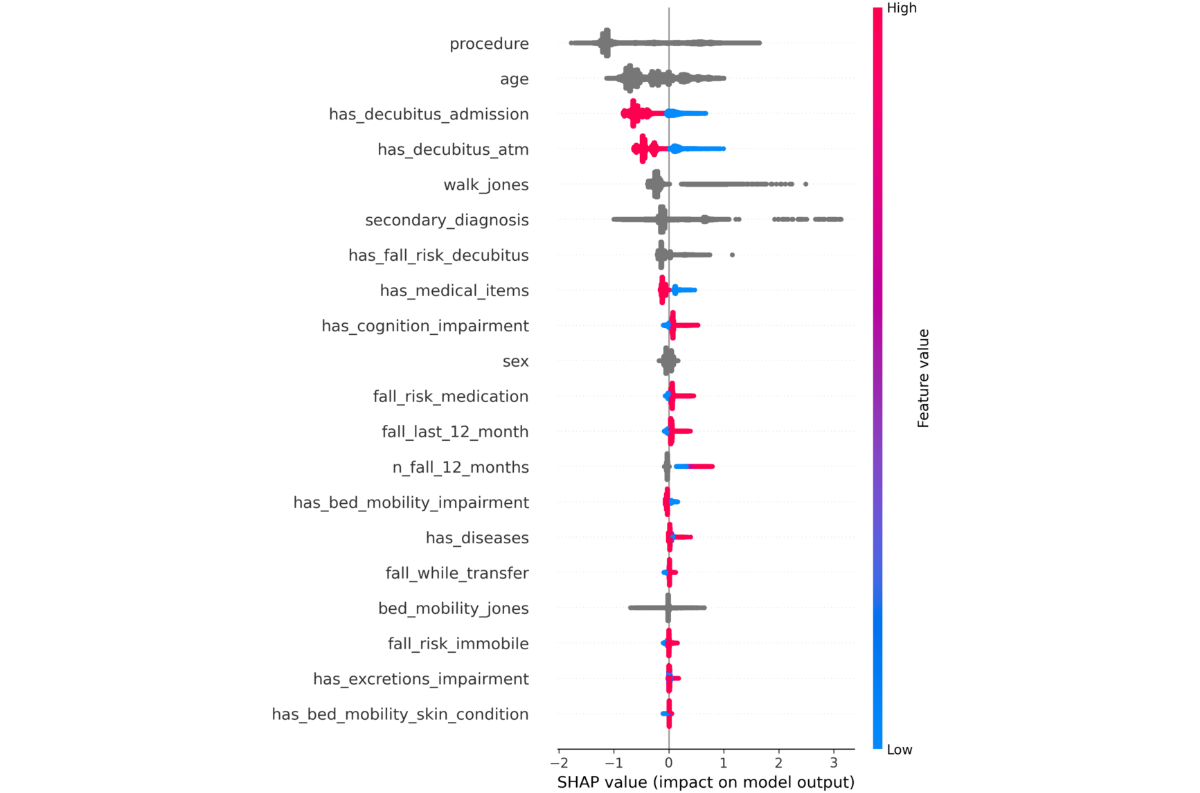
University hospital—each point along the x-axis represents a single patient and indicates its SHapley Additive exPlanations value for that feature. The SHapley Additive exPlanations values are used to determine feature importance, with features listed in descending order of their contribution to the model’s predictions. Red color denotes that the feature has a higher value, and a blue color indicates that the feature has a lower value. Categorical features are shown as gray points. The most influential features are procedure, age, has_decubitus_admission, has_decubitus_atm, the jones score, and the secondary_diagnosis. Individuals who have decubitus on admission or at the moment of the assessment are at lower risk of falling (red points along the x-axis).

To better assess the predictions of the models, we investigated the feature importances as described in the Feature Importance section for each hospital. We find that there is a notable overlap between the model-selected features and those used in clinical practice, as outlined in Fall Risk Assessments section. For the geriatric hospital, staff rely on Tinetti, TUG, and MMSE scores. The model similarly assigns high importance to tinetti and MMSE, while TUG appears in the lower half of the importance ranking (Figure 2). At the university hospital, where fall risk is evaluated using the Jones-based system, the model also identifies the walk_jones as a key feature (Figure 3). Furthermore, despite differences in dataset schemas, the models for both hospitals consistently rely on similar key features: procedure, secondary_diagnosis, age, sex, and mobility-related assessments, and tinetti for the geriatric hospital and walk_jones for the university hospital. These features consistently rank among the top 10 most important features. This alignment suggests that the models, while trained on distinct datasets, depend on comparable features.

#### Geriatric Hospital and Baseline Models

In Figure 2, we visualize the most important features for fall risk prediction from the geriatric hospital data, highlighting the influence of each feature on the model’s output. The most influential features are procedure, the billable diagnosis code (DRG), has_dementia_or_cognitive_impairment, secondary_diagnosis (as *ICD* code), age, the tinetti score, and MMSE. If a patient has dementia or cognitive impairment, they have a higher risk of falling. If a patient has a higher tinetti score, they have a lower risk of falling. In comparison to the baselines, this feature importance analysis suggests that the model overlaps with the ESFP in 4 decisive features (sex, has_dementia_or_cognitive_impairment, age, and TUG) and an overlap of 5 features with the WGFP, TUG, MMSE, barthel_index, has_delirium, and has Parkinson. This indicates that the AI models diverge in certain aspects from the rule-based models when assessing fall risk.

#### University Hospital and Baseline Models

In Figure 3, we present the most influential features for fall risk prediction from the university hospital data. The most influential features are procedure, age, has_decubitus_admission, has_decubitus_atm, walk_jones, and secondary_diagnosis (as *ICD* code). If a patient has decubitus on admission or at the moment when the assessment is done, they have a lower risk of falling. In comparison with the baselines, this feature importance analysis suggests that the model overlaps with 2 decisive features with the ESFP, age, sex, and no overlapping features with the WGFP. Again, as for the geriatric hospital, the AI models diverge from the baselines with the most decisive features.

## Discussion

### Principal Results

In this study, we compared 3 AI-based approaches for fall risk assessment with traditional rule-based systems (ESFP and WGFP) for predicting fall risk across 2 hospital datasets with considerable demographic shifts. Our results show that AI-based models consistently outperform rule-based fall risk assessment, with the university hospital dataset achieving an AUROC score of 0.926 compared with 0.703 and 0.565 for the rule-based models ESFP and WGFP models, respectively. In the geriatric hospital, the AI model similarly outperformed the rule-based models, with an AUROC of 0.735 compared with 0.556 for ESFP and 0.606 for WGFP. The improvements in recall over the rule-based models used in practice translate into more accurate identification of patients at risk for falls. The higher precision scores of the AI models mean that they could reduce the number of false positive patients if these models were used in clinical settings. This could lead to better tailored fall prevention measures. A central question in the context of AI model development in health care is how these models can be trained without compromising patients’ privacy. Developing AI models in a multicenter setting is technically challenging if data cannot be shared across institutions. We investigated popular approaches for decentralized learning such as FL and swarm learning. In our experiments, none of the decentralized learning approaches lead to improvements over the separately trained models, particularly when faced with large demographic shifts. In fact, the application of FL led to a notable drop in performance for the university hospital dataset, where the AUROC dropped from 0.926 to 0.841. In the presence of covariate shifts in data sets, a key challenge is to ensure fairness of the AI model across demographic groups. Our fairness analysis focused on 2 key metrics: predictive parity and equal opportunity across demographic subgroups, particularly age and sex. We found that the AI model predictions are fair with respect to predictive parity and equal opportunity on both datasets across the sex subgroups, but age-related disparities were more pronounced in certain subgroups. Nonetheless, the Wilcoxon signed-rank tests for age and sex subgroups did not indicate significant fairness disparities, although the test may not have reached significance due to limited sample size.

### Limitations and Future Directions

#### Challenges With FL

While FL is considered effective for collaborative model training, it did not lead to improvements in our case. Many FL algorithms are built under the assumption that the data are independent and identically distributed across clients [57,58]. When the data distribution differs across institutions, the performance of the FL model can be impacted negatively [58-60]. There are several reasons why data distributions differ across institutions and not all of them can be easily alleviated. For example, there could be demographic shifts across institutions, nursing homes, and wards. Also, there could be different pathologies and diseases more frequently expressed in individual patient populations across institutions, leading to shifts in EHR data. Other shifts are due to data infrastructure or data processing and collection. In our experiments, all the above factors could have impacted the negative results of FL. A key factor for the results could have been the sampling of the test sets. We followed the guidelines of the respective hospitals and optimized models on the respective test set of the hospital.

#### Challenges in Fairness Analysis

Finally, although our fairness analysis did not reveal statistically significant differences in performance across sex and age groups, the overestimation of fall risk in the small group of older patients should be reconsidered on a larger scale. The results of the fairness analyses depend to some extent on the specific settings, such as the bin sizes chosen for the age groups or of the definition of the majority group, which could limit the generalizability of the results.

#### Challenges With Retrospective Analyses of AI Models for Fall Risk Assessment

The limitations of retrospective analyses of AI model performance, as done in our work, are especially important to highlight in studies on fall risk. All our data were obtained from care units which carefully implemented rule-based systems motivated by national and international standards to provide the best possible risk assessment and prevention measures for their patients. As shown in our analysis, these rule-based systems tend to have a high recall. This means that they rarely assign too low fall risk to any patient truly at risk. Consequently, many patients receive therapeutic measures that effectively prevent falls. Because of these therapeutic interventions, results on the efficacy of AI models for fall risk prediction obtained in retrospective studies should be interpreted with care. And prospective studies can only include risk assessment tools that adhere to the clinical guidelines and nursing care standards.

#### Future Directions

To address our limitations, future analysis could profit from more standardized data schemas across hospitals and thus larger sample sizes collected across institutions. Since the absolute number of falls is small even in large datasets, as in this study, the pronounced label imbalance requires larger sample sizes for training and evaluating AI models for fall risk assessment. We hope that the results of our retrospective analyses will help validate AI models for fall risk prevention in prospective studies in the future.

#### Model Deployment

One of the key goals of developing fall prediction models is to support medical staff in making informed decisions. The model can be deployed as a secure web-based application, hosted on a hospital server, or on a trusted cloud infrastructure. A user-friendly interface could allow medical staff to either manually input relevant patient data or import data from the hospital’s database or patient files, like an EHR. The application can then generate a risk prediction or the probability of falling. Once a patient is entered into the database and their parameters are frequently updated, this prediction could be automatically provided, and medical staff would be timely informed, daily, or within a predefined time step.

### Comparison With Previous Work

In addition to traditional risk assessment tools, there have been attempts to develop AI models for fall risk assessment. We find our work mostly related to works of Thapa et al [3], Millet et al [4], Chu et al [28], and Tago et al [32], with which we share a common data format. However, many studies are constrained to datasets comprising only a few hundred data points, which poses significant challenges in developing models that accurately reflect real-world scenarios. In contrast to some studies [5,30,51], we keep the original data distribution. We do not remove negative samples from the distribution nor perform Synthetic Minority Over-sampling Technique to account for the imbalanced classes, in both train and test splits. In addition, most of these studies focus on data from a single institution, which limits opportunities to improve model robustness and generalizability through multi-institutional data collaboration. While these studies provide valuable insights and risk factors for fall risk prediction, they differ from our study in several key aspects. First, this study focuses on data from 2 distinct institutions that cannot be shared, aiming to train a shared AI model while retaining privacy across institutions. Second, we leverage real-world and large-scale heavily imbalanced datasets which substantially differ in their age distributions, thus exploring a more realistic scenario. Finally, none of these studies prioritize fairness metrics across demographics, whereas our goal is to investigate whether the model delivers fair predictions across all demographic groups.

### Conclusions

This study demonstrates the potential of AI models in predicting fall risk, consistently outperforming traditional expert systems across 2 hospital datasets. However, it also reveals the challenges related to demographic shifts and label distribution imbalances within the datasets, which likely negatively impacted the AI models’ ability to generalize. Our results show that training AI models in a decentralized fashion in this setting decreased their predictive performance. In addition, while the fairness analysis indicated promising predictive parity and equal opportunity across sex subgroups, our results suggest age-related disparities. To enhance model performance and fairness in future research, addressing data imbalance and ensuring broader representation across demographic groups will be crucial for developing more fair and generalizable models.

## Appendix

Multimedia Appendix 1 Additional tables and figures.

## Abbreviations

AI: artificial intelligence
AUROC: area under the receiver operating characteristic
EHR: electronic health record
ESFP: Expert Standard for Fall Prophylaxis
FDR: false discovery rate
FL: federated learning
FNR: false negative rate
ICD: International Classification of Diseases
ICD-10-GM: International Statistical Classification of Diseases, Tenth Revision, German Modification
ML: machine learning
MMSE: Mini-Mental State Examination
OPS: Operationen und Prozedurenschlüssel (Operation and Procedure Classification System)
PPV: positive predictive value
PR: precision-recall
SHAP: Shapley Additive Explanations
TUG: Timed Up and Go
WGFP: World Guidelines for Falls Prevention
XGBoost: Extreme Gradient Boosting
