# Supplementary material for: The Potential of AI in Nursing Care: Multicenter Evaluation in Fall Risk Assessment
Source: J Med Internet Res. 2025 Oct 8;27:e71034. doi: 10.2196/71034 (PMC12547331; doi:10.2196/71034)

The common data schema is presented in Table S1. Tables S2 and S3 list the parameters for the baseline models Expert Standard for Fall Prophylaxis (ESFP) and World Guidelines for Falls Prevention (WGFP) respectivelly. The results presented in the tables are derived from a 5-fold cross-validation. For each model, the evaluation metrics: F1 score, Precision (P), Recall (R) (Table S4 and S5) were calculated across the 5 folds. In (Table S6, S7, S8, S9, S10) we assess the statistical significance of the differences observed between models across various demographic groups and experimental conditions. These results were used to evaluate the models’ fairness. We measure this by comparing the positive predictive value (PPV), the false discovery rate (FDR), the false negative rate (FNR) and the true positive rate (TPR).

Table S1. Common Data Schema. id is used as index, and fall is the target column.

| Name | Type |
| --- | --- |
| id | str |
| age | category |
| sex | category |
| procedure | category |
| secondary_diagnosis | category |
| c_zriserh1 | bool |
| has_decubitus_admission | bool |
| has_decubitus_atm | bool |
| has_bed_mobility_impairment | bool |
| bed_mobility_jones | category |
| has_bed_mobility_skin_condition | bool |
| has_transfer_impairment | bool |
| has_transferred | bool |
| has_medical_items | bool |
| has_mi_arm_or_leg_splint | bool |
| has_mi_A_V_system | bool |
| has_mi_plaster_or_neck_brace | bool |
| has_mi_compression_stocking | bool |
| has_mi_ventilation_mask | bool |
| has_mi_glasses | bool |
| mobility_knee_tutor | bool |
| mobility_prosthesis | bool |
| has_mi_skin_condition_at_item_application | bool |
| fall_risk_immobile | bool |
| fall_risk_balance | bool |
| has_walking_aid | bool |
| has_excretions_impairment | bool |
| fall_risk_urge_incontinence | bool |
| fall_risk_nykturie | bool |
| has_cognition_impairment | bool |
| has_cognition_disoriented_time | bool |
| has_cognition_disoriented_location | bool |
| has_cognition_disoriented_own_person | bool |
| behavioral_pattern_excited | bool |
| has_cognition_confused | bool |
| fall_risk_medication | bool |
| has_fall_risk_decubitus | category |
| has_diseases | bool |
| n_fall_12_months | float |
| fall_last_12_month | bool |
| fall_while_transfer | bool |
| fall_while_stay | bool |
| walk_jones | category |
| nutrition_food_intake_decreased | bool |
| admission_month | float64 |
| admission_diagnosis | category |
| fall_risk_acuity | bool |
| mobility_insomnia | bool |
| barthel_stool_control | float64 |
| diabetes_oral | bool |
| fall_risk_aid | bool |
| mobilität-sonstige | category |
| tinetti_walking_test | float64 |
| behavioral_pattern_tendency_to_stray | bool |
| aid_lenses | bool |
| fall_risk_fracture | bool |
| nutrition_risk | bool |
| MMSE | float64 |
| has_depression | bool |
| barthel_stand_up_and_walking | float64 |
| aid_new_prescribed | bool |
| has_dementia_or_cognitive_impairment | bool |
| fall_risk_contracture | bool |
| sleep_apnea | bool |
| has_dizziness | bool |
| fall_risk_stool_behavior | category |
| has_calcium_deficiency | bool |
| aid_hearing_aid_left | bool |
| has_vitamin_D_deficiency | bool |
| diabetes_insulin | bool |
| private_insurance | bool |
| admission_reason | float64 |
| fall_risk_contrast_perception | bool |
| behavioral_pattern_unfocused | bool |
| mobility_no_impairment | bool |
| behavioral_pattern_appropriate | bool |
| aid_none | bool |
| main_diagnosis | category |
| tinetti | float64 |
| where_from | float64 |
| fall_risk_fear | bool |
| nutrition_low_bmi | bool |
| nutrition_critically_ill | bool |
| diabetes_no | bool |
| barthel_urine_control | float64 |
| mobility_walking_stick | bool |
| barthel_bathing_showering | float64 |
| has_delirium | bool |
| abteilung | category |
| behavioral_pattern_apathetic | bool |
| has_diabetes | bool |
| sleep_sleeping_pills | bool |
| bundesland | category |
| admission_year | float64 |
| tinetti_balance_test | float64 |
| barthel_eat | float64 |
| sleep_falling_asleep_disorder | bool |
| barthel_dressing_and_undressing | float64 |
| barthel_index | float64 |
| mobility_walking_support | bool |
| has_orthostatic_hypotension | bool |
| mobility_wheelchair | bool |
| TUG | float64 |
| aid_glass_eye_left | bool |
| fall_risk_mobility | bool |
| aid_hearing_aid_right | bool |
| has_parkinson | bool |
| barthel_sit_up_and_move | float64 |
| aid_glass_eye_right | bool |
| barthel_using_the_toilet | float64 |
| fall_risk_cognitive | category |
| fall_risk_nursing | bool |
| sleep_inconspicuous | bool |
| ward | category |
| nutrition_weight_loss | bool |
| has_overweight | bool |
| DRG | category |
| barthel_washing | float64 |
| nutrition_risk_nursing | bool |
| mobility_forefoot_shoe | bool |
| living_alone | float64 |
| barthel_climbing_stairs | float64 |
| behandlungsart | float64 |
| fall_risk_inflow_or_outflow | bool |
| fall_risk_glasses | bool |
| fall | bool |

Table S2. Columns used for Rule-based system Expert Standard for Fall Prophylaxis (ESFP).

| Name | Type |
| --- | --- |
| age | category |
| diabetes_insulin | bool |
| diabetes_oral | bool |
| nutrition_low_bmi | bool |
| sex | category |
| has_calcium_deficiency | bool |
| has_dementia_or_cognitive_impairment | bool |
| has_depression | bool |
| has_diabetes | bool |
| has_overweight | bool |
| mobility_prosthesis | bool |
| mobility_walking_stick | bool |
| mobility_walking_support | bool |
| mobility_knee_tutor | bool |
| mobility_wheelchair | bool |
| mobility_forefoot_shoe | bool |
| fall_risk_fear | bool |
| fall_risk_fracture | bool |
| fall_risk_balance | bool |
| fall_risk_contracture | bool |
| fall_risk_mobility | bool |
| TUG | float64 |

Table S3. Columns used for Rule-based system World Guidelines for Falls Prevention (WGFP).

| Name | Type |
| --- | --- |
| fall_risk_nykturie | bool |
| fall_risk_glasses | bool |
| mobility_prosthesis | bool |
| has_orthostatic_hypotension | bool |
| mobility_knee_tutor | bool |
| nutrition_risk | bool |
| aid_hearing_aid_left | bool |
| fall_risk_fear | bool |
| mobility_wheelchair | bool |
| has_vitamin_D_deficiency | bool |
| fall_risk_contrast_perception | bool |
| behavioral_pattern_excited | bool |
| mobility_walking_support | bool |
| has_parkinson | bool |
| aid_hearing_aid_right | bool |
| has_dizziness | bool |
| behavioral_pattern_tendency_to_stray | bool |
| has_depression | bool |
| TUG | float64 |
| has_delirium | bool |
| mobility_forefoot_shoe | bool |
| behavioral_pattern_unfocused | bool |
| MMSE | float64 |
| mobility_walking_stick | bool |
| fall_risk_urge_incontinence | bool |
| fall_risk_acuity | bool |
| behavioral_pattern_apathetic | bool |
| barthel_index | float64 |

Table S4. Geriatric hospital—the calibrated AI model surpasses both baselines by at least 20%. in all Macro F1, Precision and Recall. We show the median and the 90% CI, the 5^th^ and 95^th^ percentile.

|  | Separate | ESFP | WGFP |
| --- | --- | --- | --- |
| F1 | **0.63 (0.62, 0.65)** | 0.46 (0.46, 0.53) | 0.46 (0.43, 0.54) |
| R | **0.63 (0.61, 0.65)** | 0.5 (0.5, 0.54) | 0.5 (0.5, 0.56) |
| P | **0.65 (0.62, 0.66)** | 0.43 (0.43, 0.52) | 0.43 (0.43, 0.55) |
| TP | 116.0 (105.4, 133.2) | 0.0 (0.0, 64.0) | 0.0 (0.0, 200.8) |
| FN | 230.0 (212.0, 240.4) | 345.0 (281.8, 346.0) | 345.0 (145.0, 346.0) |
| FP | 198.0 (153.2, 214.8) | 1.0 (0.2, 231.0) | 0.0 (0.0, 1044.2) |
| TN | 2011.0 (1994.2, 2055.8) | 2208.0 (1978.0, 2208.8) | 2209.0 (1164.8, 2209.0) |

Table S5. University hospital—the calibrated AI model surpasses both baselines by at least 10%. in all Macro F1, Precision and Recall. We show the median and the 90% CI, the 5^th^ and 95^th^ percentile.

|  | Separate | ESFP | WGFP |
| --- | --- | --- | --- |
| F1 | **0.63 (0.62, 0.63)** | 0.39 (0.38, 0.47) | 0.5 (0.5, 0.5) |
| R | 0.62 (0.61, 0.63) | **0.7 (0.54, 0.71)** | 0.5 (0.5, 0.5) |
| P | **0.64 (0.63, 0.66)** | 0.51 (0.5, 0.51) | 0.49 (0.49, 0.49) |
| TP | 503.0 (486.8, 577.4) | 1731.0 (346.2, 1756.6) | 0.0 (0.0, 0.0) |
| FN | 1586.0 (1511.4, 1601.2) | 357.0 (332.4, 1741.8) | 2088.0 (2088.0, 2089.0) |
| FP | 1251.0 (1072.0, 1534.4) | 78593.0 (15667.0, 78856.4) | 0.0 (0.0, 0.0) |
| TN | 183006.0 (182721.8, 183185.0) | 105664.0 (105400.4, 168590.0) | 184257.0 (184256.2, 184257.0) |

Table S6. Wilcoxon signed-rank test results from comparing PPV, FDR, TPR and FNR across both gender groups from the AI (Separate) models for both geriatric and university hospital data. The analysis shows no significant differences in fairness metrics across the gender group comparisons, as p-values>0.05 for all PPV, FDR, TPR and FNR.

| Data | Group 1 | Group 2 | PPV p-value | FDR p-value | TPR p-value | FNR p-value |
| --- | --- | --- | --- | --- | --- | --- |
| University | male | female | 0.625 | 0.625 | 0.062 | 0.062 |
| Geriatric | male | female | 0.125 | 0.125 | 0.062 | 0.062 |

Table S7. Wilcoxon signed-rank test results from comparing PPV, FDR, TPR and FNR across different age groups from the AI (Separate) model trained on the geriatric hospital data. The model performance is consistent across these demographic groups (p-values>0.05). Age groups (100-110) which have only 0-values scores are excluded from the tests.

| Group 1 | Group 2 | PPV p-value | FDR p-value | TPR p-value | FNR p-value |
| --- | --- | --- | --- | --- | --- |
| 60-70 | 70-80 | 0.312 | 0.312 | 1 | 1 |
| 60-70 | 80-90 | 0.312 | 0.312 | 0.062 | 0.062 |
| 60-70 | 90-100 | 0.062 | 0.062 | 0.062 | 0.062 |
| 70-80 | 80-90 | 0.312 | 0.312 | 0.062 | 0.062 |
| 70-80 | 90-100 | 0.812 | 0.812 | 0.375 | 0.375 |
| 80-90 | 90-100 | 0.438 | 0.438 | 0.062 | 0.062 |

Table S8. Wilcoxon signed-rank test results from comparing PPV, FDR, TPR and FNR across different age groups from the AI (Separate) model trained on the university hospital. The model performance is consistent across these demographic groups (p-values>0.05). Group combinations, containing only 0 or NaN values, that could not be tested for all PPV, FDR, TPR and FNR are excluded from the table.

| Group 1 | Group 2 | PPV p-value | FDR p-value | TPR p-value | FNR p-value |
| --- | --- | --- | --- | --- | --- |
| 100-110 | 20-30 | 0.125 | 0.125 | 0.062 | 0.062 |
| 100-110 | 30-40 | 0.188 | 0.188 | 0.062 | 0.062 |
| 100-110 | 40-50 | 0.812 | 0.812 | 0.125 | 0.125 |
| 100-110 | 50-60 | 0.812 | 0.812 | 0.125 | 0.125 |
| 100-110 | 60-70 | 0.188 | 0.188 | 0.625 | 0.625 |
| 100-110 | 70-80 | 0.125 | 0.125 | 1 | 1 |
| 100-110 | 80-90 | 0.188 | 0.188 | 0.812 | 0.812 |
| 100-110 | 90-100 | 0.625 | 0.625 | 0.188 | 0.188 |
| 20-30 | 30-40 | 0.438 | 0.438 | 0.312 | 0.312 |
| 20-30 | 40-50 | 0.438 | 0.438 | 1 | 1 |
| 20-30 | 50-60 | 0.125 | 0.125 | 0.625 | 0.625 |
| 20-30 | 60-70 | 0.125 | 0.125 | 0.312 | 0.312 |
| 20-30 | 70-80 | 0.125 | 0.125 | 0.125 | 0.125 |
| 20-30 | 80-90 | 0.125 | 0.125 | 0.125 | 0.125 |
| 20-30 | 90-100 | 0.125 | 0.125 | 0.062 | 0.062 |
| 30-40 | 40-50 | 0.625 | 0.625 | 0.312 | 0.312 |
| 30-40 | 50-60 | 0.125 | 0.125 | 0.062 | 0.062 |
| 30-40 | 60-70 | 0.125 | 0.125 | 0.062 | 0.062 |
| 30-40 | 70-80 | 0.125 | 0.125 | 0.062 | 0.062 |
| 30-40 | 80-90 | 0.125 | 0.125 | 0.062 | 0.062 |
| 30-40 | 90-100 | 0.188 | 0.188 | 0.062 | 0.062 |
| 40-50 | 50-60 | 1 | 1 | 0.188 | 0.188 |
| 40-50 | 60-70 | 0.125 | 0.125 | 0.062 | 0.062 |
| 40-50 | 70-80 | 0.125 | 0.125 | 0.062 | 0.062 |
| 40-50 | 80-90 | 0.188 | 0.188 | 0.062 | 0.062 |
| 40-50 | 90-100 | 0.438 | 0.438 | 0.062 | 0.062 |
| 50-60 | 60-70 | 0.312 | 0.312 | 0.062 | 0.062 |
| 50-60 | 70-80 | 0.312 | 0.312 | 0.062 | 0.062 |
| 50-60 | 80-90 | 0.625 | 0.625 | 0.062 | 0.062 |
| 50-60 | 90-100 | 0.438 | 0.438 | 0.062 | 0.062 |
| 60-70 | 70-80 | 0.812 | 0.812 | 0.125 | 0.125 |
| 60-70 | 80-90 | 1 | 1 | 0.062 | 0.062 |
| 60-70 | 90-100 | 0.312 | 0.312 | 0.062 | 0.062 |
| 70-80 | 80-90 | 1 | 1 | 0.188 | 0.188 |
| 70-80 | 90-100 | 0.312 | 0.312 | 0.125 | 0.125 |
| 80-90 | 90-100 | 0.188 | 0.188 | 0.312 | 0.312 |

Table S9. AUROC and F1 macro scores across models performed with AutoGluon [1]: In terms of AUROC XGBoost demonstrates the best performance for the university hospital dataset, and ranks second for the geriatric hospital dataset, making it a strong candidate overall.

|  | Geriatric Hospital | | University Hospital | |
| --- | --- | --- | --- | --- |
| Model | AUROC | F1 macro | AUROC | F1 macro |
| XGBoost | 0.769 | **0.631** | **0.935** | 0.532 |
| LightGBMXT | 0.767 | 0.606 | 0.934 | 0.499 |
| CatBoost | **0.777** | 0.584 | 0.933 | 0.531 |
| LightGBM | 0.747 | 0.609 | 0.918 | 0.531 |
| LightGBMLarge | 0.753 | 0.595 | 0.917 | **0.535** |
| RandomForestEntr | 0.766 | 0.486 | 0.892 | 0.532 |
| RandomForestGini | 0.763 | 0.493 | 0.854 | 0.531 |
| ExtraTreesEntr | 0.737 | 0.471 | 0.835 | 0.499 |
| ExtraTreesGini | 0.746 | 0.471 | 0.816 | 0.499 |
| KNeighborsDist | 0.564 | 0.532 | 0.511 | 0.499 |
| KNeighborsUnif | 0.572 | 0.527 | 0.511 | 0.499 |

1. AutoGluon. Available from: https://auto.gluon.ai/stable/index.html [accessed Jul 8, 2024]

Figure S1. Fairness analysis indicates mostly fair AI fall risk assessment models, with some fairness infringements for some age groups. Geriatric hospital – (a) the AI model demonstrates a higher False Discovery Rate (FDR) for patients aged 100-110, indicating overestimation of fall risk, and a higher False Negative Rate (FNR) (b), failing to accurately identify those patients who fall. However, this fairness disparity is less pronounced in the university hospital, where the precision score is closer to parity. University hospital – (c) the AI model demonstrates lower FDR for patients aged 20-30 and 30-40, and higher FDR for patients aged 110-120 in comparison to other patients, indicating overestimation of fall risk for all other age groups. The AI model (d) is mostly fair across all groups with some exceptions in patients aged 110-120. The ratios represent the score of Group 1 over the score of Group 2. Blue shades indicate that Group 1 has a lower score than Group 2, and red shades indicate that Group 1 has a higher score than Group 2. A score of 1 indicates that both groups have equal scores. Fields between which are annotated with asterisk indicate larger disparity (a ratio lower than 0.8 or greater than 1.25). If both values in a ratio are 0, the ratio is set to 1 (parity). Empty squares indicate that the denominator (Group 2) has a zero or not available value, while the numerator (Group 1) has a non-zero value.


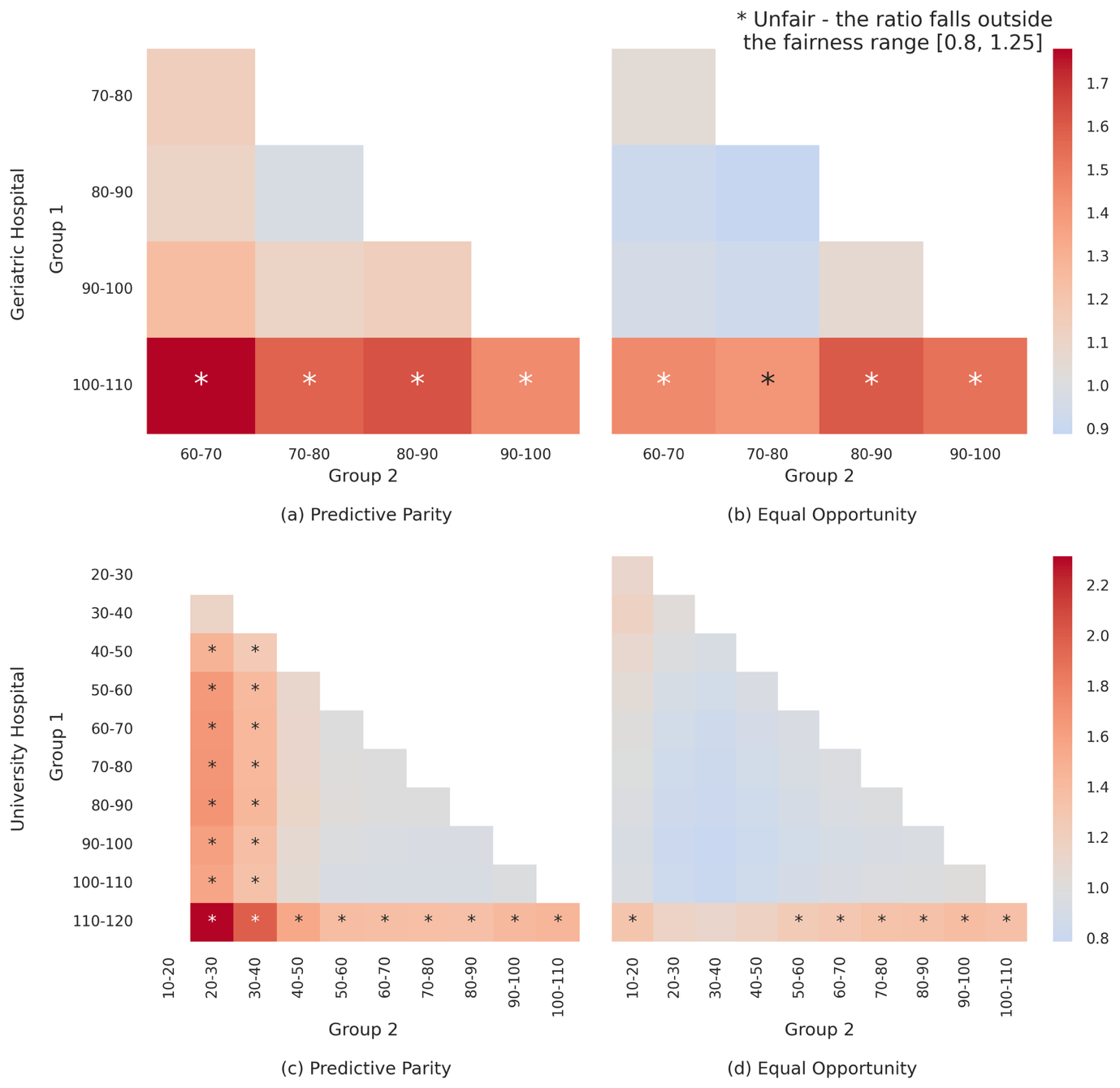

Supplement: Multimedia Appendix 1 [file jmir_v27i1e71034_app1.docx]
